# Evaluation of *Find Your Fuel*: A Point-of-Service Labelling Campaign in a Military Dining Facility

**DOI:** 10.3390/ijerph18031340

**Published:** 2021-02-02

**Authors:** Julia Carins, Francisco Crespo Casado, Sharyn Rundle-Thiele, Anna Kitunen

**Affiliations:** 1 Social Marketing at Griffith, Griffith Business School, Griffith University, Nathan, QLD 4111, Australia; s.rundle-thiele@griffith.edu.au (S.R.-T.); a.kitunen@griffith.edu.au (A.K.); 2 Defence Science & Technology Group, Land Division, Scottsdale, TAS 7260, Australia; 3 Winning Moves, Baskerville House, Birmingham B1 2ND, UK; francisco.crespo.casado@gmail.com

**Keywords:** healthy eating, nutrition, food service, labelling, evaluation

## Abstract

Nutrition plays a critical role in health and job performance in physically demanding roles. Studies have shown Australian military personnel do not consume diets suited to their roles. A range of foods are provided in military dining facilities; however, personnel still need to make appropriate choices for healthier eating and to optimise performance. This study explored the effect of a labelling scheme based on military-specific nutrition guidance, over a one-month period. Food choices were evaluated in a pre-post design using plate photography (pre *n* = 190; post *n* = 159 plates); with satisfaction and behavioural influences assessed through a survey (pre *n* = 79; post *n* = 67). The results indicate the scheme had a small effect on food choice—potato and hot vegetable choices increased post-campaign for the dinner meal. On average, choices were heathier at lunch post-campaign, and less healthy at dinner. Satisfaction with the meal experience was higher after the campaign, and no difference was observed in behavioural influences (e.g., self-efficacy and other perceptions). These results are in alignment with other point-of-service labelling studies showing the limited capacity labelling schemes have on guiding consumer choices. Rather than using point-of-service labelling in isolation, additional individual and/or environmental strategies may be needed to more effectively encourage nutritious food choices by personnel.

## 1. Introduction

Nutrition is an essential foundation for military health and performance, supporting health; weight maintenance; physical fitness; and psychological and cognitive functioning [1,2]. This is important given personnel have demanding roles involving physical and mental pressures during preparation, deployment and recovery. Three elements need to be in place to ensure consumption of a diet that supports health and performance. First, there must be an understanding of the nutritional needs of personnel; second, availability (or provision) of foods must allow for these needs; and third, personnel would need to make appropriate choices from the available foods to meet their own needs [1]. Military nutrient requirements have been described [3], providing knowledge of the nutritional needs of personnel. In Australia, these nutritional needs have been modelled to translate nutrients into whole food diets [4], which have been used to underpin guidance and contractual arrangements for the provision of foods through military dining facilities. This guidance ensures a range of suitable foods is available for personnel when they attend for meals [5]. The remaining element is the provision of assistance or guidance to enable personnel to make choices that meet their individual needs.

The intent of any point-of-service nutrition labelling scheme is to provide some assistance or guidance. Nutrition labelling is underpinned by information processing models, which assume the consumer engages in a rational process of exposure to the message, perception/attention, understanding, judgement, and behaviour [6,7,8]. This means noticing the label, understanding what the label is communicating about the food, making a judgement about the food and how it will provide a benefit or a risk, and finally making a food choice. Some models recognise dual pathways—a rational pathway (above) as well as an affective or emotional pathway, such as liking or preferences for certain foods [7,9]. To date, nutrition labelling schemes have had limited success. Numerous systematic reviews have concluded that calorie (or kilojoule) labelling does not have the desired effect on consumer choice [10,11,12,13]. Researchers have suggested that provision of contextual information (such as the recommended daily calories/kilojoules for an average adult) or interpretive information (such as traffic light labels; where green, orange, or red symbols are used to indicate appropriateness of foods) may be more effective [11,14]. However, the most effective format for nutrition labelling is yet to be determined [15], indicating further research is warranted.

In the military setting, previous point-of-service nutrition labelling that focused on calorie content or healthfulness has not been effective [16,17]. Improvements have been observed when nutrition labelling has been used as one strategy within a comprehensive intervention that also changed the food supply and food environment [18,19]. Prior studies demonstrate motivational communications (encouraging personnel to eat various food types to support performance) led to the selection of more moderately healthful foods, and more of the most healthful foods when combined with changes to the food environment [20]. In both cases, the communications were placed in the dining area, but did not label particular foods [20].

In Australia, personnel are not required to eat at the dining facility and other options such as commercial outlets can be accessed. These outlets have been shown to offer less nutritious foods, and insufficient nutrition information [21]. Delivery of an attractive food service to which personnel are willing to return can increase and maintain attendance—thereby increasing exposure to point-of-service labelling. Factors such as design, decor, layout, ambience, signs and symbols [22,23] contribute to perceptions of the dining environment. Within a dining facility, personnel experience the physical setting before they see or taste food, and a positive reaction to the setting enhances expectations for the service and food that is to come [24]. In addition to the physical dining environment, food presentation is a major influence on customer satisfaction [25]. Military dining facilities that deliver a more enjoyable experience are expected to result in higher satisfaction. Satisfaction generates repeat attendance [26], which should be a goal for outlets charged with the responsibility to deliver nutritionally balanced food offerings, given that regular attendance means diners are choosing from a healthier array of offerings.

One of the difficulties in designing nutrition labelling for military dining facilities is that personnel dining in the facility are likely to be operating at different activity categories, which may vary across the dining facility from day to day. Personnel are required to identify their activity category, then identify which foods will provide the required energy and nutrients, and assemble meals using those foods [4]. This study explored the effect of a military nutrition labelling campaign that was designed to assist personnel to identify their activity category and guide them towards foods that would provide the required nutrition for that activity category. Further, the study examined diner satisfaction to consider the extent the dining facility met or exceeded the expectations of personnel while delivering a military labelling campaign. Food choice, satisfaction and behavioural influences were measured before and after campaign implementation to determine the effect of the campaign.

## 2. Materials and Methods

### 2.1. Study Design

The ‘Find your Fuel’ campaign was implemented and tested in an Australian military dining facility in July/August 2018. The facility serves ‘other ranks’ (not top-ranking personnel). Meals are provided via a buffet style meal service offering. The buffet offers main choices (personnel are encouraged to select only one main option), and a variety of hot vegetables, salad dishes and sandwich ingredients (personnel can select as many of these as they desire). Personnel serve themselves and then move to a table of their choice to eat their meal. Cyclical menus are used within the dining facility. Although the dishes varied day to day, comparison of the menu at each time point demonstrated that similar dish types were presented in each meal (for example, consistent arrays of offerings were observed containing single meat mains such as a roast/grilled/baked meats; plus mixed meat and vegetable mains such as a stew/stir fry dishes; plus vegetarian main dishes).

The campaign was evaluated using a pre-post design, using two data collection methods. Food choice behaviour was measured using food photography; satisfaction and behavioural influences were measured using surveys; both before and after the campaign. A convenience sampling approach was employed, and a subset of personnel were intercepted by members of the research team during the meals at both time points. Personnel were approached once they had selected their meal and were asked if they would consent to a photo being taken of their plate and whether they would consider taking a survey to fill in during the meal. No identifying details (e.g., names) were collected. However, participants were requested to generate a code slip both during meal photography, and at the front of the survey, which allowed matching between photographs and surveys. Seven meals were observed before the introduction of ‘Find your Fuel’ (four lunches and three dinners over a four-day mid-week period in late July 2018), and six meals after the campaign (three lunches and three dinners over a three-day mid-week period in late August 2018). Personnel attending the facility numbers varied substantially between the pre- and post-campaign periods, being much higher prior to the campaign. This was a result of a number of personnel attending military activities away from the base during the second time point. Average attendance during lunches was 731 (pre) and 446 (post), whilst at dinner, attendance was 741 (pre) and 274 (post). Personnel were not expected to attend every meal or every day (due to work or training schedules), therefore it was expected that the composition of diners at each meal would be different. The study sought a sample size of 100 at each time point. Adequate statistical power would be provided by a sample size of 86 per group (based mean difference 0.5; SD = 1.0; 90% power).

### 2.2. Design and Implementation of the ‘Find Your Fuel’ Labelling Campaign

Point-of-service labels were designed based on Defence-specific nutrition guidance provided in the ADF EDGE (ADF Educators Guide to Healthy Eating) [4]. This guidance classifies ADF activities based on the energy requirements of military tasks and requires that personnel (1) identify an activity category; (2) identify how energy requirements might be met using serves from different food groups; and (3) construct a daily eating plan. The activity categories are shown in Table 1.

**Figure 1:**
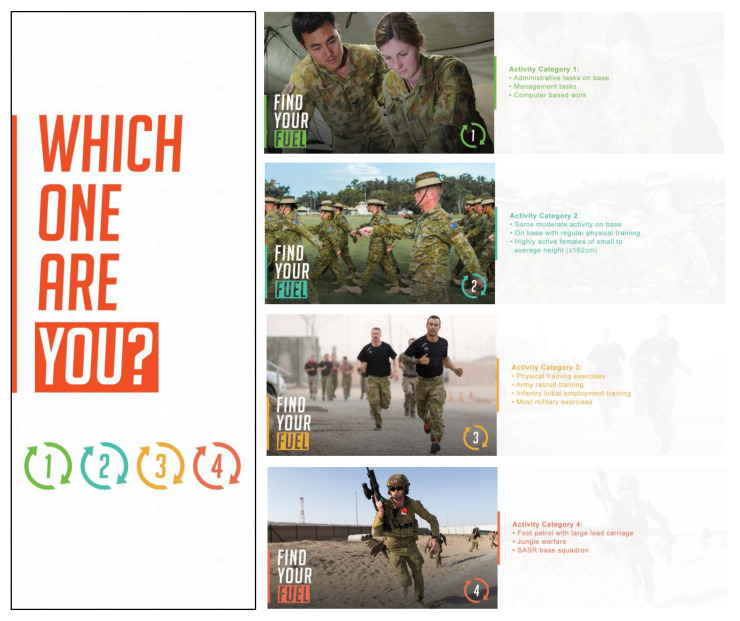
Examples of banners and cards showing activity categories.

**Figure 2:**
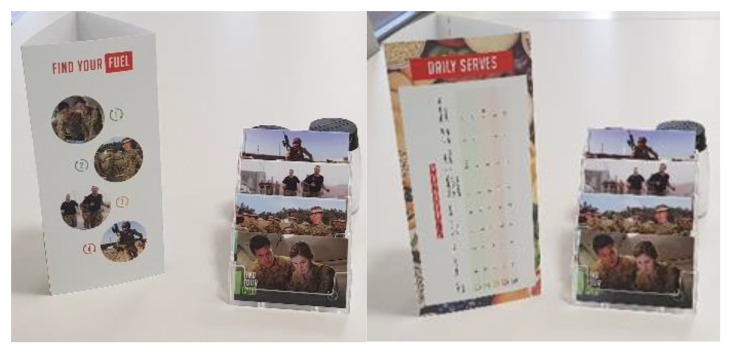
Table talkers and cards (positioned on tables).

**Figure 3:**
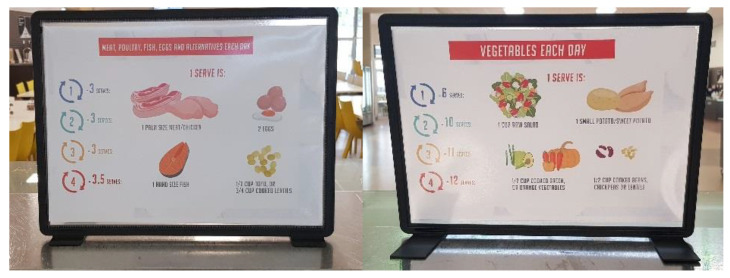
Food labels (positioned on food counters).

This facility did not service personnel undertaking the Special Air Service Regiment (SASR) selection course, so only levels 1–4 were included. The ADF EDGE details the number of food groups to be eaten per day to meet the energy requirements of each activity category and provides examples of each food group [4]. All materials were designed to adhere to this guidance, and in consultation with the catering company to ensure the feasibility of testing within one of their contracted dining facilities. The materials used to communicate the guidance were: banners, positioned to catch attention on entry to the facility; table talkers and cards, located on tables, and food labels showing the number of serves required from each food group, positioned wherever those foods were served/available. The campaign materials were installed immediately after the pre- timepoint and remained for the entire month including during the post-measurement timepoint. The campaign materials are shown in Figure 1, Figure 2 and Figure 3 below.

### 2.3. Measures

#### 2.3.1. Demographics

Demographic details for the study participants were captured in the survey using four questions for gender, length of service (years), and self-reported height (metres) and weight (kilograms). Birth year was extracted from the self-generated code to calculate the age of the respondent in years. Body mass index (BMI) was calculated for each participant using the formula weight divided by height squared.

#### 2.3.2. Survey Data Collection

The survey measured food choice influences across individual, social and environmental domains using previously validated items. Individual influences were measured by items that required respondents to nominate an optimal eating pattern from seven choices (plenty of fresh fruits, vegetables and salads; rich in complex carbohydrates; high protein; unprocessed/minimally process foods; lean meats/low fat/reduced fat foods; low sugar/sugar free, or other) and to nominate a response to several survey items in relation to that goal. Survey items measured the intention to follow that goal (three items from Perugini and Bagozzi [27]), and self-efficacy to achieve the goal (four items from Rhodes and Courneya [28]). Intention to undertake a behaviour, and self-efficacy to perform the behaviour work together to create and facilitate purposive action. Subjective nutrition knowledge, or the extent to which individuals perceive they know about nutrition, was measured as well using two different validated sets of items [29,30]. One measured subjective level of nutrition knowledge (four items from Hoefkens et al. [30]), the other measured confidence in subjective knowledge (three items from Moorman et al. [29]). Social influence was measured in terms of perceptions of what others think one ought to do (injunctive norms—three items from Norman and Conner [31]), as well as pressure to behave in the way that most others do (descriptive norms—three items from Norman and Conner [31]). Environmental influence was measured as perceptions of the environment in terms of availability of healthy foods (five items from Mujahid et al. [32]). The survey also measured one outcome, satisfaction with meals in the dining facility, using validated survey items (3 items from Carpenter [33]). All survey items (except goal nomination) were presented on seven-point Likert scales (1 to 7). Survey items are included in the Supplementary Materials.

#### 2.3.3. Food Photography

Food choice behaviour was measured using a method previously developed and validated in this context [34]. Digital plate photography is a well-developed method that has been used extensively in dietary assessment (for example see the work of Martin et al. [35]). The method involved photographing the food choices of individual diners after they had visited the food counters. In brief, personnel were approached at the point where they had finished their food selection by a researcher and were asked whether a photograph could be taken of their plate. If the diner consented to the process, a photograph was taken of the plate from above to capture as much of the food as possible for later identification. Personnel could decline having their meal photographed or take a route through the dining room to avoid the photographer (who was standing in plain sight) if they did not want their plate to be photographed.

### 2.4. Analysis

Following data entry and cleaning, descriptive and inferential statistics were estimated. Statistical analysis was conducted using IBM SPSS Statistics (Version 25) for Windows (IBM Corp, Armonk, NY, USA). Reliability of each survey construct was tested to determine whether the individual survey items were capturing the intended underlying constructs using Cronbach’s alpha. Reliability was considered acceptable when Cronbach’s alpha met or exceeded the 0.70 threshold [36,37]. Construct scores were calculated as the average of items comprising that construct. Comparisons between construct scores were conducted using independent samples t-tests, with significance determined at *p* < 0.05. Each plate photograph was independently examined by two analysts who matched food selections on each plate to the list of dishes available for that meal. The number of differences in identification between the two analysts was less than 5% of the total number of identifications indicating close agreement. The choices on each plate were grouped into mains; vegetables (including potato dishes, rice/pasta dishes and hot vegetables); salads; and sandwich choices. Choices were classified as red (least healthful), orange (moderately healthful) and green (most healthful) according to a previously developed classification scheme [34]. Comparisons between choices in these categories were conducted using independent samples t-tests, with significance determined at *p* < 0.05.

## 3. Results

### 3.1. Sample Description

During the pre-campaign data collection period, 79 surveys were collected, and 190 photographs taken. At post-campaign, 67 questionnaires were collected, and 159 meals were photographed. On average this means around 5% of meals were photographed, a similar capture rate to other studies conducted in this setting [20,34]. Many personnel who had consented to having their meal photographed declined the opportunity to complete a survey. The survey results indicate the pre- and post-campaign groups were very similar in terms of demographics (see Table 2 below), however it must be noted that the surveys are a sub-sample of those who consented to a plate photograph.

### 3.2. Survey Results—Influences on Food Choice

#### Comparison Pre- and Post-Communication Campaign

Almost all constructs were found to be reliable, with Cronbach’s alpha approaching or exceeding the cut-off (α > 0.70) [36,37]. The construct of descriptive norms was below the cut off (α = 0.63), but for the purposes of this study was considered marginally acceptable, given the items have been shown to be reliable in previous studies (see Table 3 below).

All constructs were above the midpoint of 4 prior to campaign suggesting these behavioural influences were high (e.g., respondents reported relatively high subjective nutrition knowledge). No significant differences were observed between constructs measured pre- and post-campaign. Surveys were not completed by all participants, reducing analytical power. However, it is important to note that differences of 0.5 between means were not observed for any behavioural influence construct (see Table 3 below).

### 3.3. Food Photography—Food Choice Behaviour

Three photographs were excluded from the 190 photos taken during the pre-campaign point due to food items being obscured from view, making it impossible to account for all choices on those plates. Of the remaining photographs, 117 were taken at lunch, and 70 at dinner. At the post-campaign point, five photographs were removed (food items obscured) leaving 93 lunch and 61 dinner photographs. Meals typically consisted of one main choice, and up to six choices from the vegetable, salad and sandwich ingredient choices. Of these sides, two or three vegetable choices were typically chosen (including potato dishes, rice/pasta dishes and hot vegetables), along with one sandwich ingredient, and one salad choice. Differences between meal photographs taken pre- and post-campaign are shown in Table 4, based on averages for each meal (lunch and dinner).

Few differences were observed when comparing pre- and post-campaign plate photographs. Total number of selections on plates remained stable (M pre = 5.8, M post = 5.9; *t*(339) = −1.004, *p* = 0.316). In terms of food choice types, differences in the number of main meal dishes is not expected, as most personnel choose to take a main choice, but are discouraged from taking a second main choice. However, variations in the other dish types can reasonably be expected. For dinner meals, the average number of choices from the hot side dish category was significantly higher post-campaign, with a mean difference of 0.5. This difference represents one in two diners taking an extra hot side dish choice post-campaign. When examining this change in more detail, increases in two of the three hot side dish sub-groups was noted—potato (0.3 mean increase) and vegetables (0.34 mean increase), with no difference in the third subgroup (rice/pasta). In real terms these differences indicate one in three people selected an extra hot vegetable dish or potato dish (not both) post-campaign. Vegetable consumption could occur via salad or hot vegetables, and when selections from these categories were combined (salads plus potato plus vegetables) a significant mean increase of 0.47 was observed—almost one in two people selected an extra vegetable containing side dish post-campaign at the dinner meal.

In terms of health categories, the number of red choices was lower after the campaign at lunch, and the number of orange choices was higher, with no change in green choices (see Table 5). This represents a change in average selections within the dining facility in a positive direction. At dinner, this trend was reversed, the number of red choices was higher after the campaign, and the number of orange choices was lower, again with no change in green choices. This represents a negative change in average selections. When meals were analysed together, the number of orange choices increased, but the number of red and green choices remained similar.

### 3.4. Survey Results—Satisfaction

Satisfaction with the meal experience was measured, with survey items that were found to be reliable (Cronbach’s α = 0.94). An independent samples t-test was conducted to compare satisfaction pre- and post-campaign. Mean satisfaction was above the scale midpoint of 4 prior to campaign (mean satisfaction = 4.75) and was significantly higher post-campaign (mean satisfaction = 5.22). This indicates the personnel surveyed post-campaign expressed more satisfaction with the meal experience they had in the dining facility that those surveyed pre-campaign (*t*(142.6) = −2.051, *p* = 0.042).

## 4. Discussion

Contractual arrangements for provision of foods through military dining facilities [5] are designed to ensure that a nutritious range of foods is available for personnel. Personnel are free to choose from the range of foods within this setting, and presently no nutrition information schemes are routinely used to assist personnel to make healthier food choices. This study implemented and evaluated the ‘Find your Fuel’ campaign at a military dining facility, a point-of-service labelling scheme designed to assist personnel to select foods suited to their individual activity category.

The results indicate the scheme had a small effect on food choices, with personnel selecting more from the counters containing hot vegetables—in particular, potato dishes and hot vegetable dishes. There were no observable changes in choices for mains (to be expected), salads or sandwich fillings. This is encouraging, as several studies have found that Australian soldiers consume diets that are low in carbohydrate and high in fat (especially saturated fat-rich) [38,39,40]. Increased consumption of potato and vegetable dishes will increase carbohydrate consumption and aid personnel to meet recommended consumption levels for vegetables. However, the trend in the healthfulness of choices was mixed—at lunch positive changes were noted, and at dinner, negative changes. This may indicate a change in food choices by personnel towards meeting energy requirements (increased carbohydrate intake) but not always through selection of the most healthful alternatives (for example, through dishes that are higher in fat and salt). These differences between lunch and dinner may be reflective of different food preferences at each meal. Preferences for lighter meals at lunch and heavy, pleasurable meals in the evening [41,42] may mean that hedonic preferences are more influential than nutrition guidance at certain times of the day. The impact of healthier eating at one meal, but not another, would need to be determined and this offers a rich avenue to explore in future studies.

Satisfaction with the dining experience was observed to be higher post-campaign. Personnel often have negative expectations or experiences with military provided foods [43,44], considering them to be less acceptable [45], and as a result many choose to dine elsewhere. However, commercial outlets surrounding bases frequently provide a lower level of support for healthful eating [21]. The objective for feeding within military dining facilities should be to provide nutritionally balanced and appealing food, with high levels of satisfaction, so that dining in the facility becomes the preferred option for personnel. Although the food choices personnel made were largely unaltered in this study, they expressed a higher level of satisfaction with the dining experience post-campaign. This implies there were no adverse effects from the campaign on the dining experience and may indicate the labelling was appealing and appropriate. Recent studies evaluating military dining facility improvement efforts involving changes to the physical space, to food presentation and introducing new food options have resulted in improved satisfaction ratings [46].

No differences were observed for the behavioural influences measured in this study. Ecological models of behaviour posit that both individual factors (such as intentions, knowledge, and self-efficacy) and environmental influences (social, such as norms; and physical, such as availability) determine behaviour [47]. These findings suggest that the labelling did not impact these behavioural influences. We did not expect that labelling would produce strong and wide-ranging effects given these were not the direct focus of the labelling only campaign. It is important to capture behavioural influence measures to build an evidence base to inform future behavioural change campaign efforts.

This study indicates that a military-specific point-of-service labelling scheme, based on Defence specific nutrition guidance [4], had limited effect on food choice. Whilst this may be considered a disappointing outcome, it indicates that more needs to be done. Reviews highlighting the limited success of labelling schemes have focused on identification of alternative, more effective, labelling formats [10,11,12,13]. However, evidence indicates that programs that move beyond information provision and communication are, in fact, more effective [18,19,20,48]. Interventions that address multiple levels (individual, social and environmental levels) are considered to be most effective in promoting healthful behaviour [47]. In addition, social marketing frameworks recommend a mix of strategies to bring about behaviour change [49], utilising individual approaches to motivate behaviour change in combination with modifications to the surrounding environment to support behaviour. In line with these recommendations, follow-up studies are needed to determine the effect of a broader suite of strategies to encourage healthier eating behaviour in military dining facilities.

### Strengths and Limitations

A strength of this work is evaluation of the campaign in a real setting, combined with assessment of food choice using plate photography. These two aspects overcome some of the challenges presented by evaluation under laboratory conditions and self-report methodologies. However, the study contains limitations which represent avenues for future research. Firstly, this evaluation used a cross-sectional design. As individuals may not attend every meal or every day, longitudinal studies are needed to measure the impact on individuals who are repeatedly exposed to the campaign. A longitudinal design that includes follow up at a later timepoint is needed to determine the impact of labelling over the longer term. Future longitudinal studies should examine group differences considering women are often more motivated to improve dietary behaviour and improve nutrition knowledge [50,51]. Increasing the size or representativeness of the plate photography sample would give more confidence that the sample is representative of the total dining population. This study was conducted in one facility, replication in other dining facilities servicing different military populations would provide the ability to draw definitive conclusions. The use of activity categories is a novel concept, however, future evaluations should include methods to determine which category personnel belong to, whether they can recognise which category they belong to, and how they translate that understanding into food selections. Measuring personnel’s perceptions and preferences for the campaign materials would be an important addition to support refinement of the labelling concept. Finally, the addition of strategies that increase knowledge, intentions and self-efficacy is likely to support effective use of point-of-service labelling and would further assist personnel to improve food choices.

## 5. Conclusions

Nutrition plays a critical role in military health and performance. Military feeding in dining facilities is underpinned by guidance designed to provide a range of nutritious foods. Personnel are then required to choose foods to meet their nutrition requirements. This study found that a point-of-service labelling scheme had a small effect on food choice. This aligns with other point-of-service labelling studies showing labelling has minimal impact on consumer choices. Therefore, rather than using point-of-service labelling in isolation within military dining environments, additional individual and/or environmental strategies may be needed to more effectively encourage nutritious food choices by personnel. Strategies tested should be based on best practice principles for behaviour change and involve consideration of an ecological model of behaviour (motivating individuals and creating supportive environments).

## Figures and Tables

**Table 1:** ADF Activity Categories (from [4] p. 23).

Number	Activity Category	Energy Range	Example Activities
1	Inactive or sedentary	<12.5 MJ/day (3000 Kcal/day)	Administrative tasks on base
2	Light activity	13–14.5 MJ/day (3100–3500 Kcal/day)	Navy ships or submarine at sea, some moderate activity on base
3	Moderate activity	15–16.5 MJ/day (3600–4000 Kcal/day)	Physical training exercises, Army recruit training, Infantry initial training, most other military exercises
4	High activity	17–21 MJ/day ^1^ (4000–5000 Mcal/day)	Foot patrol with large load carriage, jungle warfare, naval clearance diving, patrol boat, SASR base squadron
5	Extreme activity	≥21 MJ/day (≥5000 Kcal/day)	SASR ^2^ selection course

^1^ Two category 4 energy ranges are merged into one category 4 range in this table. ^2^ SASR: Special Air Service Regiment. ADF: Australian Defence Force.

**Table 2:** Demographics for survey participants: pre- and post-communication (pre *n* = 79; post *n* = 67 surveys).

Demographics	Pre-Communication Campaign		Post-Communication Campaign	
	Count	Mean (Std Dev)	Count	Mean (Std Dev)
Male	69	-	58	-
Female	4	-	5	-
Other	2	-	1	-
(missing)	4	-	3	-
Age (years)	-	26 (5.3)	-	26 (7.0)
Service years	-	4.5 (3.8)	-	4.9 (4.2)
BMI ^1^	-	25.8 (2.8)	-	25.3 (2.6)

^1^ One BMI result of 42 was removed from analysis (possibly a self-report error of height/weight).

**Table 3:** Survey measures: pre (*n* = 79) and post (*n* = 67) communication campaign.

Survey Construct	α	Pre		Post		*t*-Test	
(7-Point Scales)		*n*	Mean	*n*	Mean	T (df)	*p*
Intention	0.79	78	5.10	67	5.22	−0.65 (135)	0.515
Nutrition knowledge ^1^	0.77	78	5.50	67	5.71	−1.31 (143)	0.192
Nutrition knowledge ^2^	0.82	77	5.44	66	5.66	−1.38 (141)	0.170
Self-efficacy	0.88	78	5.46	67	5.76	−1.67 (143)	0.098
Descriptive norms	0.63	78	4.53	67	4.86	−1.83 (143)	0.069
Injunctive norms	0.78	78	5.34	67	5.68	−1.91 (143)	0.058
Perceived availability	0.89	78	4.51	67	4.83	−1.59 (143)	0.113

^1^ Level of nutrition knowledge (subjective); ^2^ Confidence in subjective nutrition knowledge.

**Table 4:** Selections from each food category: pre- and post-communication (pre *n* = 190; post *n* = 159 meal photos) ^1^.

Meal	Number of Selections		Pre-		Post-		*t*-Test	
	By Food Category	By Sub-Category	N	Mean	N	Mean	T (df)	*p*
Lunch	Main		117	1.00	93	0.99	0.65 (208)	0.519
	Hot side dishes		117	2.59	93	2.80	−1.45 (205)	0.149
		Potato	117	0.90	93	0.89	0.08 (208)	0.936
		Rice/pasta	117	0.31	93	0.40	−1.36 (192)	0.175
		Vegetables	117	1.38	93	1.51	−1.02 (208)	0.309
	Salad		117	0.67	93	0.77	−0.96 (208)	0.340
	Sandwich ingredients		117	1.20	93	1.09	0.59 (208)	0.558
	(Salad + Potato + Vegetables)		117	2.95	93	3.17	−1.53 (208)	0.127
Dinner	Main		70	0.99	61	1.00	−0.93 (129)	0.353
	Hot side dishes		70	2.27	61	2.77	−3.00 (129)	0.003
		Potato	70	0.63	61	0.92	−3.70 (126)	<0.001
		Rice/pasta	70	0.44	61	0.31	1.55 (128)	0.123
		Vegetables	70	1.20	61	1.54	−2.13 (113)	0.035
	Salad		70	0.54	61	0.38	1.63 (129)	0.106
	Sandwich ingredients		70	1.20	61	0.95	1.10 (129)	0.274
	(Salad + Potato + Vegetables)		70	2.37	61	2.84	−2.56 (158)	0.012

^1^ Eight photos (3 pre; 5 post) were removed from analysis (food items were obscured from view).

**Table 5:** Selections from each healthfulness category: pre- and post-communication (pre *n* = 190; post *n* = 159 meal photos) ^1^.

Meal	Number of Selections from Each Healthfulness Category	Pre-		Post-		*t*-Test	
		N	Mean	N	Mean	T (df)	*p*
Lunch	Red choices	117	1.68	93	1.27	3.24 (208)	0.001
	Orange choices	117	1.30	93	2.22	−6.72 (208)	<0.001
	Green choices	117	3.02	93	2.69	1.55 (208)	0.122
Dinner	Red choices	70	0.74	61	1.74	−7.64 (129)	<0.001
	Orange choices	70	2.07	61	1.31	4.26 (129)	<0.001
	Green choices	70	2.69	61	2.62	2.39 (129)	0.811
Lunch &	Red choices	187	1.33	154	1.47	−1.40 (339)	0.164
Dinner	Orange choices	187	1.59	154	1.86	−2.30 (339)	0.022
(combined)	Green choices	187	2.89	154	2.66	1.40 (339)	0.163

^1^ Eight photos (3 pre; 5 post) were removed from analysis (food items were obscured from view).

## Data Availability

The data presented in this study are available on request from the corresponding author. The data are not publicly available due to privacy and ethical reasons.

## Supplementary Materials

The following are available online at https://www.mdpi.com/1660-4601/18/3/1340/s1, Survey Measures—All 7-Point Scales
