# Supplementary material for: Evaluation of Find Your Fuel: A Point-of-Service Labelling Campaign in a Military Dining Facility
Source: Int J Environ Res Public Health. 2021 Feb 2;18(3):1340. doi: 10.3390/ijerph18031340 (PMC7908192; doi:10.3390/ijerph18031340)
Supplement: Supplementary file 1 [file ijerph-18-01340-s001.pdf]

SUPPLEMENTARY MATERIALS – SURVEY MEASURES – ALL 7-POINT SCALES
[NB: **Defence** is the organisational name of the Australian military; and personnel call a military dining facility a **Mess**]

Intention (adapted from Perugini & Bagozzi, 2001).
I am planning to [*follow a diet that helps me perform my Defence role*] during the next 2 weeks
I will expend effort to [*follow a diet that helps me perform my Defence role*] during the next 2 weeks
I intend to [*follow a diet that helps me perform my Defence role*] during the next 2 weeks

Self-efficacy (adapted from Rhodes & Courneya, 2003)
How confident are you that you will be able to [*follow a diet that helps you perform your Defence role*] in the next 2 weeks?
How confident are you over the next 2 weeks that you could overcome obstacles that prevent you from [*following a diet that helps you perform your Defence role*]?
How confident are you that you could [*follow a diet that helps you perform your Defence role*] over the next 2 weeks, if you wanted to?
I believe I have the ability to [*follow a diet that helps me perform my Defence role*] over the next 2 weeks

Subjective Nutrition Knowledge (from Moorman *et al*, 2004)
How do you rate your nutrition knowledge compared to the average person?
How do you rate your confidence in using nutrition information compared to the average person?
Do you feel confident in your ability to comprehend nutrition information on product labels?

Subjective Nutrition Knowledge (from Hoefkens *et al*, 2012)
I have a lot of knowledge about how to prepare a healthy meal
I know which foods are healthy for me
I have a lot of knowledge about how to evaluate the nutritional value of a food
Those who know me consider me to be an expert in healthy foods

Injunctive Norms (adapted from Norman & Conner, 2006)
People who are important to me want me to [*follow a diet that helps me perform my Defence role*]
People who are important to me would approve of me [*following a diet that helps me perform my Defence role*]
People who are important to me think I should [*follow a diet that helps me perform my Defence role*]

Descriptive Norms (adapted from Norman & Conner, 2006)
Do you think your friends [*follow a diet similar to one that would help you perform your Defence role*]?
Do you think your family members [*follow a diet similar to one that would help you perform your Defence role*]?
Do you think others in this Mess [*follow a diet similar to one that would help you perform your Defence role*]?

Perceived Availability (adapted from Mujahid et al., 2007)
The fresh fruits and vegetables [*in this Mess*] are of high quality
A large selection of fresh fruits and vegetables is available in [*this Mess*]
[*A large selection of protein foods is available in this Mess*]
A large selection of low-fat products is available in [*this Mess*]
[*A large selection of energy rich products is available in this Mess*]

Satisfaction (adapted from Carpenter, 2008)
I am pleased with [*the dining experience I had in the Mess today*]
I am happy with [*the dining experience I had in this Mess today*]
I am contented with [*the dining experience I had in this Mess today*]
